# Important roles of P2Y receptors in the inflammation and cancer of digestive system

**DOI:** 10.18632/oncotarget.7518

**Published:** 2016-02-19

**Authors:** Han-Xing Wan, Jian-Hong Hu, Rei Xie, Shi-Ming Yang, Hui Dong

**Affiliations:** ^1^ Department of Gastroenterology, Xinqiao Hospital, Third Military Medical University, Chongqing, P.R. China; ^2^ Division of Gastroenterology, Department of Medicine, School of Medicine, University of California, San Diego, California, USA

**Keywords:** P2Y receptors, digestive inflammation, digestive cancer

## Abstract

Purinergic signaling is important for many biological processes in humans. Purinoceptors P2Y are widely distributed in human digestive system and different subtypes of P2Y receptors mediate different physiological functions from metabolism, proliferation, differentiation to apoptosis *etc*. The P2Y receptors are essential in many gastrointestinal functions and also involve in the occurrence of some digestive diseases. Since different subtypes of P2Y receptors are present on the same cell of digestive organs, varying subtypes of P2Y receptors may have opposite or synergetic functions on the same cell. Recently, growing lines of evidence strongly suggest the involvement of P2Y receptors in the pathogenesis of several digestive diseases. In this review, we will focus on their important roles in the development of digestive inflammation and cancer. We anticipate that as the special subtypes of P2Y receptors are studied in depth, specific modulators for them will have good potentials to become promising new drugs to treat human digestive diseases in the near future.

## INTRODUCTION

Purinoceptors are generally divided into P1 receptors which main ligand is adenosine, and P2 receptors which main ligands are nucleotides. Based on their signaling properties, P2 receptors are further subdivided into ionotropic P2X receptors that are nucleotide-gated ion channels and metabotropic P2Y receptors that are G protein-coupled receptors (GPCRs). P2Y receptors are consist of eight subtypes: five Gq/G11-coupled subtypes (P2Y1, P2Y2, P2Y4, P2Y6 and P2Y11), usually activating phospholipase C-IP_3_ pathway that modulates endoplasmic reticulum calcium release, and three Gi/o-coupled subtypes (P2Y12, P2Y13 and P2Y14), mainly inhibiting adenylyl cyclase to regulate cyclic AMP/protein kinase A (PKA) [1–2]. At present, all eight P2Y receptor subtypes have been cloned in mammalian [3]. Different P2Y receptor subtypes are activated by different nucleotides. ADP has been claimed to be selective agonist of P2Y1, P2Y12 and P2Y13 receptors; however, UTP predominantly binds to P2Y2 and P2Y4 receptors, and to a lesser extent to P2Y6 receptors which preferential agonist is UDP. P2Y14 receptors are mainly activated by UDP-glucose and other UDP-sugars, or by UDP [4]. In recent decades, a growing line of evidence suggests the involvements of P2Y receptors in the pathogenesis of human diseases, and different subtypes of P2Y receptors mediate various pathophysiological processes, ranging from metabolism, proliferation, differentiation to apoptosis. Recent studies also demonstrate that P2Y receptors play important roles in the regulation of physiological functions and pathological processes in the digestive system. In this review, we will focus on the pathophysiological roles of P2Y receptors in digestive inflammation and cancer.

## PHYSIOLOGICAL FUNCTIONS OF P2Y RECEPTORS IN THE DIGESTIVE ORGANS

**Figure 1:**
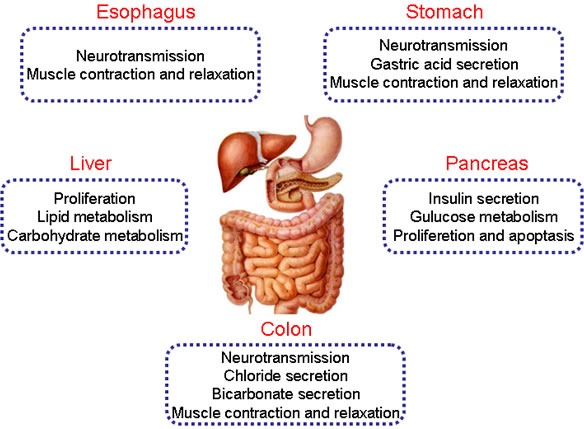
The physiological functions of P2Y receptors in digestive system Different subtypes of P2Y receptors are expressed in human esophagus, stomach, liver, pancreas and colon. They play different roles in the regulation of physiological processes, such as neurotransmission, ion transports, metabolism, proliferation and apoptosis, muscle contraction and relaxation in the digestive organs.

P2Y receptors are widely expressed in digestive organs and their functions vary from neurotransmission, gland secretion, contraction and relaxation of smooth muscle to carbohydrate and lipid metabolism in the digestive system. In accordance with evidence, we first highlight physiological roles of P2Y receptors in the esophagus, stomach, liver, pancreas, and colon (Figure 1).

### Esophagus

The P2Y receptors are functionally expressed in the esophagus to play an important role in the regulation of esophageal motility. In human and porcine esophagus, P2Y1 receptors mediate lower esophageal sphincter (LES) relaxation by regulating neurotransmission [5–6]. P2Y1 receptors also mediate contraction of the circular smooth muscle layer in porcine esophagus [7]. Electrical field stimulation (EFS)-induced contractions is mediated through P2Y receptors in cat esophageal smooth muscle [8]. Feline esophageal contraction is preferentially mediated by P2Y receptors coupled to Gαi_3_ and Gαq proteins, which activate PLCβ, subsequently increase intracellular Ca^2+^ and activate PKC [9].

### Stomach

Although gastric acid secretion is mainly regulated by P1 adenosine receptors [10], P2Y receptors may also regulate gastric acid secretion, gastric contraction, relaxation and neurotransmission. However, the specific receptor subtypes of P2Y receptors involved and their underlying mechanisms still need further investigation. ATP selectively inhibits histamine-stimulated gastric acid secretion from rabbit parietal cells by acting on P2Y receptors [11]. UTP and UDP can induce contraction of gastric smooth muscle through activation of P2Y receptors [12]. ATP also induces contraction of gastric smooth muscle in guinea pig *via* activation of P2Y receptors [13]. At least two subtypes of P2Y purinoceptors are involved in gastric contraction in guinea-pig and are related to the elevation of intracellular Ca^2+^ [14]. Finally, ATP may regulate NANC inhibitory neurotransmission in rat pyloric sphincter through acting on P2Y receptors [15].

### Liver

Several P2Y receptor subtypes regulate hepatic physiological functions, such as carbohydrate metabolism, lipid metabolism and proliferation [16–17]. P2Y1 receptors induce glycogen phosphorylase of rat hepatocyte by raising intracellular calcium concentrations but inhibiting cyclic AMP accumulations [18]. P2Y2 receptors in human hepatocytes regulate both glycogen metabolism and proliferation-associated responses through Ca^2+^ and MAPK pathways [19], and induce ERK phosphorylation, Egr-1 expression, and cyclins and cell cycle progression, which are essential for efficient hepatocyte proliferation [20]. P2Y2 receptors also mediate extracellular ATP-induced c-jun N-terminal kinase signaling and cell cycle progression to promote hepatocellular proliferation [21]. P2Y13 receptors modulate reverse cholesterol transport by increasing hepatic HDL cholesterol uptake, overall hepatocyte cholesterol content, and biliary output [22–24].

### Pancreas

P2Y1, 2, 4, 6, 11, 12 and 13 receptor subtypes have been identified in INS-1βcells, mouse, rat and human pancreaticβcells [25–26]. Activation of P2Y receptors inβcells is confirmed to participate in the regulation of insulin secretion, glucose metabolism and an increase in intracellular Ca^2+^ concentration [27]. Glucose stimulation triggers exocytosis of insulin and ATP through activation of P2Y1 receptors to result in PLC activation and DAG production in MIN6 mouse pancreaticβcells [28]. P2Y1 and P2Y6 receptors in MIN6 cells induce intracellular calcium release and insulin secretion, and prevent TNF-α induced βcells apoptosis [29]. Stimulation of P2Y13 receptors in pancreatic βcells inhibits insulin secretion *via* calcium influx and inhibition of cyclic AMP production [30]. High glucose and free fatty acids can also induce β cells apoptosis through stimulating P2Y13 to activate apoptotic pathways [31].

### Colon

Large numbers of studies in animals and humans demonstrate that P2Y1 receptors mediate NANC inhibitory transmission of intestinal smooth muscle in mice through release of ATP and NO [10]. Activation of P2Y1 receptors can induce nerve-mediated relaxation *via* inhibitory neuromuscular transmission in human intestines, guinea pig small intestine and rat colon [32–35]. Endogenous nucleotides acting on P2Y1, 2, 4 receptors evoke intestinal Cl^−^ secretion [36]. The activation of P2Y2 and P2Y4 receptors stimulate Cl^−^ secretion in small and large intestines. Basolateral UTP-induced Cl^−^ secretion in jejunum was partially reduced in P2Y2 knockout (40%) and P2Y4 knockout (60%) null mice [37]. Activation of P2Y2 receptors induces duodenal mucosal bicarbonate secretion *via* both intracellular Ca^2+^ release and extracellular Ca^2+^ entry through store-operated channels [38]. Stimulation of luminal P2Y2 and P2Y4 receptors lead to K^+^ secretion in mouse distal colonic mucosa [39]; however, activation of basolateral P2Y6 receptors on rat colonic enterocytes induces NaCl secretion *via* a synergistic increase of [Ca^2+^]_i_ and cyclic AMP [40].

## P2Y RECEPTORS IN DIGESTIVE INFLAMMATION

Over the past decades, many studies have highlighted fundamental roles of P2Y receptors in inflammatory diseases, particularly P2Y2, 6, 12 receptors have been well studied, such as P2Y2 receptor agonists can treat cystic fiborosis, and promote wound healing and leukocyte functions [4, 41–45]. Whereas, P2Y2 receptors have ambivalent functions, such as promoting chronic inflammatory states and fibrotic remodeling [46–48]. However, in inflammation of the digestive system, the studies of P2Y receptors are mainly concentrated on the liver and colon. The involvements of P2Y receptors in the inflammation of other digestive organs, such as esophagitis, gastritis and pancreatitis, are poorly understood and rarely presented in the literatures.

### Liver

During inflammatory responses, endogenous release of ATP in the liver can activate purinergic P2 receptors. It was found that large amounts of ATP released from the liver increased the expression of P2Y2 receptors in concanavalin A-induced model of acute hepatitis in C57BL/6 mice. Liver damage and necrosis are largely decreased in C57BL/6 wild-type mice injected with suramin, an inhibitor of P2Y receptors, or in P2Y2 receptors knockout mice, in which acetaminophen-induced liver damage is also alleviated. P2Y2 receptors can promote neutrophil infiltration, regulate cell survival, and promote tumor necrosis factor-mediated cell death, supporting the view that activation of P2Y2 receptors stimulates the recruitment of neutrophils into the liver to cause hepatocyte death [49].

Hepatic stellate cells play an important role in formation of liver fibrosis and liver cirrhosis. ATP increases intracellular Ca^2+^ in hepatic stellate cells, which is inhibited by suramin. Interestingly, P2Y2 and P2Y4 receptors are expressed in quiescent hepatic stellate cells, whereas P2Y6 receptors are expressed in activated hepatic stellate cells. The activated hepatic stellate cells express the ectonucleotidase nucleoside triphosphate diphosphohydrolase-2 (NTPDase-2) that colocalizes with activated HSC in CCl_4_-induced cirrhosis. UDP regulates transcription of procollagen-1 in activated HSC *via* P2Y receptors activation, which is partially inhibited by the P2Y receptors inhibitor suramin, suggesting P2Y receptors may be attractive targets to prevent/treat liver fibrosis [50]. When macrophages in kupffer cells of the liver are activated by various factors, such as lipopolysaccharide (LPS), they produce various cytokines and chemokines to play important roles in hepatitis and liver fibrosis. P2Y2, 5, 6, 12, 13 receptors are strongly expressed in the liver kupffer cells of C57BL/6 mice (KUP5 cells). After stimulation with LPS, KUP5 cells produce IL-6 and TNF-α. Non-selective P2 receptor antagonist, suramin, and P2Y13 receptors selective antagonist, MRS2211, markedly inhibit LPS-induced IL-6 increase in KUP5 cells, whereas both suramin and MRS2211 do not inhibit LPS-induced TNF-α production, suggesting that P2Y13 and other P2Y receptors may be involved in LPS-induced IL-6 production in Kuffer cells and liver inflammation [51].

### Colon

Intestinal inflammation can upregulate mRNA expression of P2Y2 and P2Y6 receptors in the colonic mucosa of colitic mice. The mRNA of P2Y2 and P2Y6 receptors are increased in both Crohn's and ulcerative colitis of intestinal human samples compared with noninflamed tissues. The mRNA expression of P2Y2 receptors is also increased in Caco-2 and IEC-6 cells during intestinal inflammation, but it is unknown how inflammation up-regulates expression of P2Y2 receptors. ATP or UTP stimulation of P2Y2 receptors in intestinal epithelial cells increases ICAM-1 expression and promotes transepithelial migration and adhesion of neutrophils and macrophage to the apical surface of IEC. In addition, ATP or UTP facilitates the migration of neutrophil-like PLB-985 cells and macrophage across the Caco-2 monolayer and promotes macrophage-like U-937 cells adhere to IEC monolayers. CD68+ macrophage infiltrates from the colonic epithelium and presents at the apical surface of colonocytes during intestinal inflammation. P2Y2 receptors mediate neutrophil adhesion to the surface of IEC need presence of adherent macrophage. This investigation is helpful to identify potential therapeutic targets to treat inflammatory bowel diseases [52]. Recent studies found that NF-κB p65 could regulate P2Y2 receptors transcription, and activation of P2Y2 receptors by UTP increased both cyclooxygenase-2 (COX-2) expression and PGE2 release in IEC [53]. Further studies showed the effects of CCAAT/enhancer-binding proteinβ (C/EBPβ) and NF-κB p65 on P2Y2 receptor transcription are synergistic during inflammation in IEC [54]. In the enteric nervous system, ATP has long been established as an inhibitory neurotransmitter, 75% of Hirschsprung's disease patients, aganglionosis is confined to the intestine. The expression of P2Y1 and P2Y2 receptors are absent from the submucosal and myenteric plexuses of aganglionic tissue compared to ganglionic tissue and normal controls. The deficiency of P2Y receptors in ganglionic intestine in Hirschsprung's disease suggests the absence of the inhibitory neurotransmitter, ATP. This explains the contracted state of the aganglionic gut in Hirschsprung's disease [55].

The expression of P2Y6 receptors is enhanced by inflammation with TNF-α and IFN-γ both in IEC-6 and Caco-2/15 cells. In Caco-2/15 cells, stimulation P2Y6 receptors by UDP results in an increased expression and release of CXCL8, partially depending on ERK1/2 phosphorylation. UDP also increases ERK1/2 phosphorylation of IEC-6 cells, suggesting the involvement of P2Y6 receptors [56]. Indeed, UDP stimulation of P2Y6 receptors promotes CXCL8 transcription through ERK1/2 activation and the AP-1 complex of transcription factors, aggravating colitis-like disease in mice by stimulating neutrophil recruitment at the site of inflammation. CXLC8 gene expression is regulated at the transcriptional level by mediating ERK1/2-dependent phosphorylation of c-fos in IEC through P2Y6 receptors activation. P2Y6 regulation of CXCL8 expression requires PKCδactivation upstream of the signaling pathway composed of MEK1/2-ERK1/2 and c-fos [57]. Previous reports revealed that T cells play an important role in the pathogenesis of IBD and extracellular nucleotides can regulate colonic epithelial cell damage during inflammation. Interestingly, UDP, P2Y6 receptors selective agonist, activates peripheral T cells and increases mRNA levels of P2Y6 receptors, and raises intracellular calcium concentration. Although P2Y6 receptors are expressed in human T cell infiltrating IBD, the roles of P2Y6 receptors in the pathogenesis of IBD need further studies [58].

## P2Y RECEPTORS IN DIGESTIVE CANCER

**Figure 2:**
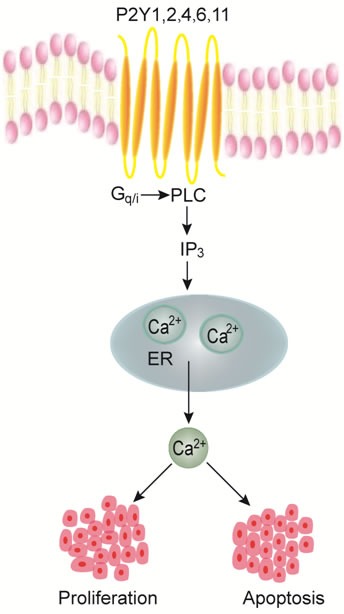
P2Y receptors-mediated Ca^2+^ signaling in proliferation or apoptosis of digestive cancer cells Stimulation of Gq/G11-coupled P2Y receptor subtypes (P2Y1, P2Y2, P2Y4, P2Y6 and P2Y11) activates PLC/IP_3_ pathway to induce intracellular calcium release from the endoplasmic reticulum (ER). An increase in intracellular calcium concentrations would increase the proliferation or apoptosis of different digestive cancer cells.

Different subtypes of P2Y receptors are expressed in many cancer cells and tissues to be likely involved in cancer development, such as P2Y receptors in melanoma [59], skin squamous cell carcinoma [60], lung cancer [61–62], prostate cancer [63–66], glioma [67–68], breast cancer [69–75], ovarian cancer [76], and haematological malignancies [77], etc. Recent growing lines of evidence suggest an important role of P2Y receptors in digestive tumorigenesis. Different subtypes of P2Y receptors are present in cancer cells and primary cancer tissues of digestive system; however, the mechanisms by which these receptors play in the devolvement and progression of cancers are still poorly understood. The involvement of P2Y receptors in digestive cancer is mainly investigated in esophageal cancer, hepatocellular carcinoma, biliary cancer, pancreatic cancer and colorectal cancer, which are summarized in Table 1 and Figure 2. Although expression and function of P2Y receptors are well documented in normal human and animal stomach, their involvements in the pathogenesis of gastric cancer have not been explored so far.

**Table 1:** Involvement of P2Y receptors in various types of digestive cancer

Cancer types	Tissue cell line	P2Y receptor subtypes	Signaling pathway	Pathological mechanisms	Reference
Esophageal cancer	Kyse-140	P2Y2	PLC/Ca^2+^	anti-proliferative apoptosis-inducing	[78]
Hepatic carcinoma	Huh-7 Rat hapatoma cell line	P2Y1,2,13	Ca^2+^	volume-regulatory cell metabolism	[79–81]
	HePG2 BEL-7404	P2Y2	Ca^2+^	promoting proliferation migration growth	[82]
	Rat hepatoma HTC cells	P2Y2	Ca^2+^ MAPK	glucose metabolism	[83]
	HePG2 huh-7	P2Y1,2,4,6	Ca^2+^	unknown	[84]
Biliary cancer	Mz-Cha-1	P2Y1,2,4,6	Ca^2+^	unknown	[85]
Pancreatic cancer	PANC-1	P2Y1,2, 6	PLC IP_3_/PKC	pro-proliferative	[86–87]
Colon cancer	HT-29	P2Y2	ECAR	tumor cell metabolism	[90]
	HT-29 calo320MD	P2Y2	Ca^2+^ cyclic AMP	anti-proliferative apoptosis-inducing	[91]
	HT-29 Primary cancer	P2Y2,4	Ca^2+^	anti-proliferative apoptosis-inducing	[92–93]
	HCT8 Caco2	P2Y1,2,4,6,11,12	Ca^2+^	pro-proliferative apoptosis-inducing	[94]
	HT-29	P2Y2	ERK P38 MAPK	anti-proliferative apoptosis-inducing	[95–96]
	Caco2	P2Y2,4	Ca^2+^ MAPK	proliferative	[97–99]

### Esophageal cancer

The squamous esophageal cancer cell line, Kyse-140 cells express mRNA of P2X4, P2X5, and P2Y2 receptors, but not P2X1 and P2X7 receptors that are mainly associated with apoptosis. The mRNA of P2Y2 and P2X4 receptors are also found in biopsies of human squamous esophageal cancer; however, in accordance with the Kyse-140 cells, the mRNA of the P2X1 and P2X7 receptors are not expressed in human squamous esophageal cancer biopsy. Although mRNA of P2Y2 and P2X4 receptors was found in both Kyse-140 and esophageal primary culture cells, only P2Y2 receptors protein specific fluorescence was detected in the membrane of the Kyse-140 and esophageal primary culture cells. ATP, UTP and ATPγS, responding to P2Y2 receptors activation, result in an increase of intracellular Ca^2+^ levels. Incubation of Kyse-140 cells with the phospholipase C inhibitor U73122 dose-dependently inhibits ATP-induced intracellular Ca^2+^ level, suggesting that P2Y2 receptors mediate intracellular Ca^2+^ *via* phospholipase C (PLC) activation in Kyse-140 cells. Extracellular ATP as well as ATP analogue dose-dependently increase the proportion of cells in the S-phase of the cell cycle in return inhibiting proliferation of primary cell cultures of human esophageal cancer as well as Kyse-140; however, only ATP but not ATP analogue dose-dependently induces caspase-3 activity and increases in apoptosis of Kyse-140 cells through activation of P2Y2 receptors [78]. Together, these findings suggest that purinergic nucleotides may be tumor preventers through P2Y2 receptors/PLC/Ca^2+^ signaling in squamous esophageal cancer.

### Hepatocellular carcinoma

P2Y1 purinergic receptors were found to play a role in the response of hepatocellular carcinoma (HCC) cells to osmotic swelling and involved in the volume-regulatory response [79]. However, in HCC cells, copper inhibits thapsigargin-sensitive Ca^2+^ stores by acting P2Y2 receptors to lead to inhibition of regulatory volume decrease (RVD) [80]. The mRNA and protein levels of P2Y13 receptors are confirmed in Huh-7 hepatoma cells that can release ATP when exposed to hypotonicity medium. On the other hand, ADP can activate P2Y13 receptors to potentiate volume regulatory decrease (RVD) and then mediate cell metabolism [81].

A recent study showed that both mRNA and protein expression levels of P2Y2 receptors were dramatically higher in native human HCC and human HCC cell lines compared with human normal hepatocytes. Extracellular nucleotides-induced intracellular Ca^2+^ increase is markedly higher in human HCC cells than normal hepatocytes. Activation of P2Y2 receptors significantly promotes proliferation and migration of HCC cells and volume growth of HCC in nude mice through store-operated calcium channels (SOCs)-mediated Ca^2+^ signaling [82]. Insulin and ATP induce a dose-dependent increase in p44/42 MAPK phosphorylation in rat HCC cells and chelation of extracellular Ca^2+^ with EGTA diminishes ATP- and insulin-induced p44/42 MAPK phosphorylation. Patch clamp electrophysiology and fluorescence microscopy showed that insulin and ATP induced monophasic and multiphasic changes in membrane potential and intracellular Ca^2+^ in HCC cells. Therefore, insulin and ATP effects are synergistic to regulate glucose metabolism of HCC cells [83]. Although the mRNA of P2Y1, P2Y2, P2Y4 and P2Y6 receptors were detected in HepG2 and HuH-7 cells, P2Y1 and P2Y6 receptor agonists, ATP and UDP, did not alter intracellular Ca^2+^, suggesting that these receptors are not expressed at functional levels. However, UTP through activation of P2Y2 and P2Y4 receptors can mobilize internal Ca^2+^ *via* inositol 1,4,5-trisphosphate (IP_3_) [84]. Therefore, these P2Y receptors may play major roles in the pathogenesis of HCC.

### Biliary cancer

Although the mRNAs for P2Y1, P2Y2, P2Y4 and P2Y6 purinergic receptors subtypes are found in biliary epithelial cancer cells (Mz-Cha-1), but only P2Y2 receptors are present at the protein level. Not only extracellular ATP dose-dependently results in an intracellular Ca^2+^ increase, but also UTP produces a similar Ca^2+^ response and cross-desensitation. ATP induces cytosolic and nuclear Ca^2+^ transients [85]. To date, only expression of P2Y receptors is observed in biliary epithelial cancer cells, however, the roles of P2Y receptors in biliary cancer need further investigation.

### Pancreatic cancer

P2Y receptors, especially P2Y1, P2Y2 and P2Y6 receptors are highly expressed in PANC-1, a duct epithelial cell derived from human primary pancreatic cancer cells. P2Y1 and P2Y6 proteins were also found in PANC-1 cells. ADP activation of P2Y1 receptors and UDP acting P2Y6 receptors increase proliferation of PANC-1 cell through PLC/IP_3_/PKC pathway. This proliferative action of P2Y receptors may potentially apply to recover pancreatic duct epithelial damage by physiological or pathological processes [86]. UTP or P2Y2 receptor selective agonist MRS2768 can increase proliferation of PANC-1, which is significantly decreased by P2Y receptor antagonist suramin and siRNA against P2Y2 receptors. UTP/P2Y2 receptor regulation of pancreatic cell proliferation depends on PLC/IP_3_/PKC and phosphorylation of Akt [87]. In INS-1 cells and rat pancreatic islets, ATP at low concentrations increases insulin release *via* P2Y receptors and PLC; however, ATP at high concentrations inhibits insulin release after metabolizing to adenosine [88]. So far, the roles of P2Y receptors in pancreatic cancer are poorly understood and need lucubrating.

### Colorectal cancer

P2Y2 and P2Y4 receptors are overexpressed in human colon cancer compared with normal colon tissues although their functional significance need further studies [89]. Immunocytochemistry and western blot analysis also demonstrate the protein expression of P2Y2 receptors in HT-29 human colon carcinoma cells. ATP or UTP elicits a biphasic effect of extracellular acidification rate by activating P2Y2 receptors in HT-29 cells, but effects of UTP or ATP are resistant to suramin, suggesting that agonists of purinoceptors may affect tumor cell metabolism [90]. The mRNA of P2Y2 receptors is expressed in two colorectal carcinoma cell lines (HT29, Colo320DM) and short-term stimulation of P2Y2 receptors cause both intracellular Ca^2+^ release and transmembrane Ca^2+^ influx, and a subsequent increase in cyclic AMP. This effect is inhibited by BAPTA-AM. Prolonged stimulation of P2Y2 receptors induces a time-dependent increase in apoptosis in both cell lines and causes a dose-dependent inhibition of cell proliferation up to 85% (Colo320 DM) or 64% (HT29). Chelating [Ca^2+^]_i_ with BAPTA-AM almost completely abolishes this effect. Moreover, forskolin or cyclic AMP derivatives cause a rise in intracellular cyclic AMP and lead to synergistic anti-proliferative effect in both cell lines. This finding demonstrates P2Y2 receptors play major roles in anti-proliferative and apoptosis-inducing in colorectal carcinoma cell lines [91]. The primary cell cultures of human colorectal carcinomas and HT29 cell line express functional P2U-receptors (P2Y2 and P2Y4). ATP or UTP at micromolar concentrations leads to a rapid biphasic increase of [Ca^2+^]_i_ and cross-desensitization between two nucleotides. P2U-receptor agonist ATP derivative ATP-γ-S inhibits proliferation and induces apoptosis of HT 29 cells [92–93].

Two human colorectal carcinoma cell lines (HCT8 and Caco-2) express mRNA of P2Y1, 2, 4, 6, 11, 12 receptors and proteins of P2Y1 and P2Y2 receptors. ATP, at high concentrations, induces apoptosis through P2Y1 receptors; conversely, ATP, at lower concentrations, and UTP stimulates proliferation of human colorectal carcinoma cells, probably acting on P2Y2 receptors. UTP can trigger calcium influxes through either P2Y2 or P2Y4 receptors, which is inhibited by suramin. Therefore, stimulation of purinergic receptors may contribute to the modulation of epithelial carcinoma cell proliferation and apoptosis [94]. Ursolic acid could inhibit proliferation of HT29 and induce apoptosis *via* P2Y2 receptors-mediated inhibition of ERK phosphorylation and activation of p38 MAPK pathway [95–96]. The mRNA of P2Y2 and P2Y4 receptors is found in Caco-2 cells. ATP, UTP and UDP increase phosphorylation of MAPK by stimulating P2Y receptors, probably through subtypes of P2Y2, P2Y4, P2Y6 and P2Y11 receptors. ATP increases proliferation of Caco-2 cells *via* activation of P2Y purinergic receptors [97–98]. On the contrary, higher concentrations (1-10 mM) of extracellular ATP or the unhydrolyzed ATP analogue 5′-adenylyimido-diphosphate (AMP-PNP), suppress Caco-2 cell proliferation arresting cells cycling at the S phase by inhibiting PKC, ERK and MAP kinase [99]. The sustained activation of P2 receptors by ATP may lead to IL-8 secretion from human colorectal epithelial cells and may play an important role in tumor progression as well as in the pathology of IBD [100].

## CONCLUSIONS

Growing lines of evidence suggest that P2Y receptors are involved in inflammation-associated diseases of liver and colon. P2Y receptors can also regulate metabolism, proliferation, differentiation and apoptosis of digestive cancer cells and tissues. It has been demonstrated that different P2Y receptor subtypes are present on the same cell and that various subtypes of receptors may produce opposite functions. Such as HCT8 and Caco-2 cells express P2Y1, P2Y2, P2Y4, P2Y6, P2Y11, and P2Y12 receptors. Lower concentrations of ATP and UTP stimulate proliferation of these cells *via* P2Y2 receptors activation, but high concentrations of ATP induce apoptosis and anti-proliferation through P2Y1 and P2X7 receptors. This suggests that the control of cell proliferation by extracellular nucleotides might be regulated by a crucial balance of the activities of the receptor subtypes. The subtypes of P2Y receptors as new therapeutic targets for drug discovery to treat digestive diseases may have extensive clinical significance. We therefore anticipate that as these subtypes of P2Y receptors in digestive organs are further studied, their specific modulators may become promising new drugs to treat digestive diseases, such as inflammation and cancer in the near future.

## Abbreviations

GPCRs: G protein-coupled receptors
PKA: protein kinase A
LES: lower esophageal sphincter
NTPDase-2: nucleoside triphosphate diphosphohydrolase-2
LPS: lipopolysaccharide
COX-2: cyclooxygenase-2
IBD: inflammatory bowel diseases
RVD: volume regulatory decrease
SOCs: store-operated calcium channels
Mz- Cha-1: biliary epithelial cancer cells
AMP-PNPATP: analogue 5′-adenylyimido-diphosphate
HCC: hepatocellular carcinoma
PLC: phospholipase C
IP3: inositol 1,4,5-trisphosphate
